# Time since onset might be of essence: A recommendation to assess the effects of combination of non-pharmacological neuromodulatory approaches at early stage since symptoms onset

**DOI:** 10.3389/fneur.2023.1115370

**Published:** 2023-01-30

**Authors:** Mariana Agostinho, Irit Weissman Fogel, Roi Treister

**Affiliations:** ^1^The Cheryl Spencer Department of Nursing, Faculty of Social Welfare and Health Sciences, University of Haifa, Haifa, Israel; ^2^Centre for Interdisciplinary Health Research, CIIS, Institute of Health Sciences, Universidade Católica Portuguesa, Lisbon, Portugal; ^3^Physical Therapy Department, Faculty of Social Welfare and Health Sciences, University of Haifa, Haifa, Israel

**Keywords:** neuromodulation, non-invasive brain stimulation, combined therapy, analgesic therapy, mirror therapy

## Abstract

In the past decade researchers began to assess the potential beneficial effects of non-invasive brain stimulation (NIBS) combined with a behavioral task as a treatment approach for various medical conditions. Transcranial direct current stimulation (tDCS) applied to the motor cortex combined with another treatment approach has been assessed as analgesic treatment in neuropathic and non-neuropathic pain conditions, and was found to exert only modest pain relief. Our group results show that combined tDCS and mirror therapy dramatically reduced acute phantom limb pain intensity with long-lasting effects, potentially preventing pain chronification. A review of the scientific literature indicates that our approach differs from that of others: We applied the intervention at the acute stage of the disease, whereas other studies applied the intervention in patients whose disease had already been established. We suggest that the timing of administration of the combined intervention is critical. Unlike in patients with chronic painful condition, in which the maladaptive plasticity associated with pain chronification and chronicity is well-consolidated, early treatment at the acute pain stage may be more successful in counterbalancing the not-yet consolidated maladaptive plasticity. We encourage the research community to test our hypothesis, both in the treatment of pain, and beyond.

## 1. Introduction

### 1.1. Transcranial direct current stimulation (tDCS) for the treatment of pain

Although the use of electrical currents for medical treatment has been documented historically (1–3), technological developments in recent decades have enabled the use of electrical-based non-invasive brain stimulation techniques, such as transcranial magnetic stimulation and transcranial direct current stimulation (tDCS), to alleviate various symptoms, such as depression and pain. This perspective article focuses on the combination of tDCS plus an additional non-pharmacological neuromodulatory treatment aimed at relieving pain.

tDCS is believed to exert its effects by modulating the resting membrane potential of a neuron and thereby changing the threshold for generating action potentials (4). Anodal motor cortex stimulation is a common montage often tested for the treatment of pain. The analgesic effect of anodal tDCS of the motor cortex was proposed to originate from local and connectional effects in remote cortical and subcortical areas through enhanced neuronal excitability. Current evidence suggests that M1 stimulation modulates thalamic and somatosensory activity by descending corticothalamic pathways, brain areas of the fronto-striatal circuit, limbic brain areas, and the periaqueductal gray [i.e., (4–6)].

### 1.2. Combining tDCS with other non-pharmacological neuromodulatory approaches

Although the past 20 years have seen much research on the effects of tDCS on both the brain and pain (7), the accumulated results of the early investigations highlighted only modest and short-term analgesic effects. More recently, researchers hypothesized that combining tDCS with another neuromodulatory treatment could enhance analgesic effects (7–11).

To address this hypothesis, researchers began to explore the analgesic effects of such combined treatments in various pain indications, including phantom limb pain (12–14), neuropathic pain (15–23), complex regional pain syndrome (24, 25), fibromyalgia (26–33), headache (34), chronic musculoskeletal pain (35), chronic low-back pain (36–40), knee osteoarthritis pain (41–45), temporomandibular disorders (46), burning mouth syndrome (47), chronic visceral pain (48), neurogenic pain (49), myofascial pain (50, 51), tendinopathy (52), and radiculopathy (53) (Table 1).

**Table 1:** Painful indications and the neuromodulatory approaches used in combination with transcranial direct current stimulation (tDCS).

**Painful indications**	**Neuromodulatory approaches**
• Phantom limb (12–14) • Neuropathic pain due to traumatic brachial plexus injury (15) • Spinal cord injury (16–22) • Complex regional pain syndrome (24, 25) • Fibromyalgia (26–33) • Chronic musculoskeletal pain (35) • Chronic low-back pain (36–40) • Knee osteoarthritis pain (41–45) • Temporomandibular disorders (46) • Chronic visceral pain (48) • Neurogenic pain (49) • Myofascial pain (50, 51) • Tendinopathy (52) • Radiculopathy (53) • Burning mouth syndrome (47) • Headache/migraine (34)	• Mirror therapy (12–15) • Visual illusion (16–18, 22) • Motor graded imagery (24) • Exercise (20, 26, 27, 33, 36, 41, 46, 47, 51, 52, 54) • Physical therapy (25, 28, 34, 35, 37–39, 43, 45, 48–50, 53, 55, 56) • Cognitive and behavioral interventions (21, 29, 30, 32, 40, 42, 47, 57)

The other neuromodulatory approaches that were combined with the tDCS could be grouped into 4 categories: The first category includes mirror therapy (12–15), visual illusion (16–18, 22) and motor graded imagery (24). These three interventions are sharing similar characteristic—in all these behavioral tasks the participants receive (or imagine) visual input (with, or without additional sensory-motor input) that is assumed to counterbalance the maladaptive plasticity associated with the painful condition. The second category of neuromodulatory approaches includes different exercises (20, 26, 27, 33, 36, 41, 46, 47, 51, 52, 54), in which participants were requested to use a treadmill to perform aerobic exercise or to produce a series of movements specifically intended to increase mobilization, strength and endurance of a painful limb. The therapeutic effects of these exercises are assumed to be produced *via* modulation of several systems, such as enhancement of corticothalamic excitability, and motor and attentional areas, increase in activity of the descending pain modulatory system and release of dopaminergic and endogenous opioids (58–60). The third category of neuromodulatory approaches comprised of other physical therapy interventions, included the use of transcutaneous electrical nerve stimulation, intramuscular electrical stimulation, mobilization through physical therapy, among other similar techniques, (25, 28, 34, 35, 37–39, 43, 45, 48–50, 53, 55, 56). These approaches assumed to activate descending pain inhibition systems and promote the release of endogenous opioid mechanisms (45, 61–63). The fourth category includes cognitive/behavioral interventions, in which participants perform cognitive tasks such as attentional, memory, executive functioning tasks, mindfulness-meditation, or breathing interventions which are also related to attention processes, processes that are commonly impaired in chronic pain patients (21, 29, 30, 32, 40, 42, 47, 57). These tasks target brain regions such as dorsolateral prefrontal cortex and limbic brain areas, that process cognitive and emotional demands of painful stimuli and exerts a role in modulating pain perception and related emotions (64–70). Summary of all neuromodulatory interventions that were assessed in conjunction with tDCS for the treatment of pain are summarized in Table 1.

### 1.3. Combined treatment at early stage of the painful condition

In a paper published by our group (12), we compared the effects of mirror therapy stand alone or with either real or sham tDCS on phantom limb pain. The study included 30 lower limb amputees who had been amputated up to 8 weeks previously and who were in the acute phase of phantom pain. Participants were randomized into 1 of the 3 groups (mirror therapy, mirror therapy + sham tDCS, mirror therapy + real tDCS) receiving 10 sessions (5 per week). They were assessed at baseline, at the end of the intervention, and 1 and 3 months thereafter, with the change in pain intensity between baseline and 1 month following the end of treatment predefined as the primary end-point.

**Figure 1:**
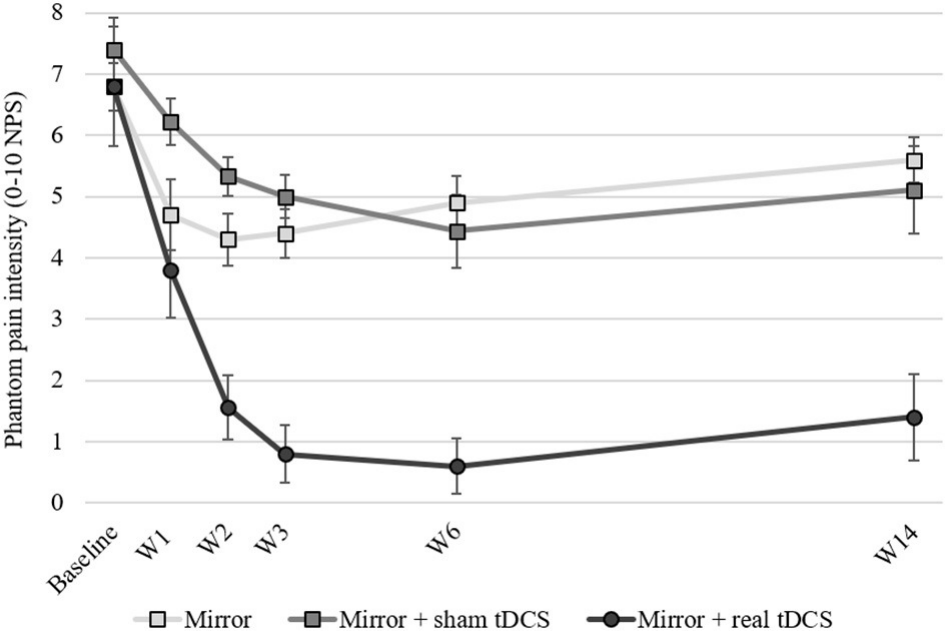
Phantom pain intensity at baseline, across treatment weeks and up to 3 months following the end of treatment. W1, during 1st week of treatment; W2, during 2nd week of treatment; W3, 1st week following end of treatment; W6, 1-month following the end of treatment; W14, 3-months following the end of treatment. tDCS, transcranial direct current stimulation; NPS, numerical pain scale. Error bars represent the Standard Error of the Mean (SEM).

The analgesic effects seen in our study were overwhelming (Figure 1). 3 months after the end of the treatment, the combined-treatment group experienced a robust analgesic effect, with mean pain reduction of 5.4 ± 2.6 points (on a 0–10 scale), and in percentage of change, about an 80% reduction), significantly more than the other 2 study arms. The analgesic effects were so large that it virtually eliminated the development of chronic phantom pain, with 90 and 80% of participants reporting pain of ≤2/10 at 1 and 3 months after the end of treatment, respectively. The analgesic effects in the two control arms were, in line with the literature, only modest, leaving the participants with significant phantom pain (>5/10) 3 months after the end of treatment.

## 2. Hypothesis

While most methodological aspects of our study were identical or similar to all the other studies that tested the effects of tDCS combined with other neuromodulatory therapy, there was one clear distinction: our study was the only one in which the patients were at the acute stage of pain. Hence, the unprecedented huge analgesic effects seen in our study might be attributed to this characteristic—the short time between the onset of the phantom limb pain and the administration of the therapy. All the other studies included chronic pain patients—that is, those who had been experiencing pain for a long time, sometimes even years or decades.

To gain more insight on our hypothesis, we searched the literature for all relevant studies that used similar treatment approaches, including mirror therapy, visual illusion, and motor graded imagery combined with tDCS. We summarized the relevant studies results in Table 2. To support a fair comparison, only studies in which 10 treatment sessions (or more) were administrated were included in the table. The indications included in the table consist of phantom pain, spinal cord injury, neuropathic pain due to traumatic brachial plexus injury, and complex regional pain syndrome. While our study included only participants who were amputated < 8 weeks previously, all the other studies included only patients with chronic pain. Treatment characteristics were similar: All the studies except ours used anodal motor cortex stimulation at 2 mA. Our study used 1.5 mA in an attempt to support blinding. To compare the clinical effects of adding tDCS to the other therapy, we gathered the means (and standard deviations) of pain scores before (at baseline) and after each study arm. Whenever possible (not all studies included the two relevant study arms), we calculated the analgesic effects in terms of standardized effect sizes (Cohen's d), as follows: the change in pain in the combined treatment (real tDCS plus real other intervention) minus the change in pain in the sham tDCS plus real other intervention, divided by their pooled standard deviation.

**Table 2:** Comparison of the analgesic effects among similar studies of tDCS combined with other therapies for pain.

**Study**	**Authors**	**Pain indication**	**Time since onset**	**Study arms (*N*)**	**Number of treatment sessions**	**Baseline pain intensity (mean ± SD)**	**Pain intensity 1 month following end of treatment (mean ± SD)**	**Change in pain following treatment (mean ± SD)**	**Effect size (Cohen's d)**
1	Segal et al. (12)	Phantom pain after unilateral lower limb amputation	<8 weeks	Mirror therapy (10)	10	6.80 ± 1.23	4.90 ± 1.37	−1.9 ± 1.30	
				Sham tDCS and Mirror therapy (10)	10	7.40 ± 1.65	4.44 ± 1.88	−2.96 ± 1.77	1.58
				Real tDCS and mirror therapy (9)	10	6.80 ± 2.94	0.60 ± 1.35	−6.2 ± 2.29	
2	Gunduz et al. (13)	Phantom pain after unilateral lower limb amputation	≥3 months	Sham tDCS and sham mirror therapy (27)	10 sham tDCS plus 20 sham mirror therapy sessions; first 10 sessions were combined	5.90 ± 1.57	3.31 ± 2.57	−2.59 ± 2.13	
				Real tDCS and sham mirror therapy (28)	10 real tDCS plus 20 sham mirror therapy sessions; first 10 sessions were combined	6.29 ± 1.67	2.93 ± 2.65	−3.36 ± 2.21	
				Sham tDCS and mirror therapy (28)	10 sham tDCS plus 20 real mirror therapy sessions; first 10 sessions were combined	6.03 ± 1.75	4.25 ± 2.55	−1.78 ± 2.19	0.47
				Real tDCS and mirror therapy (29)	10 real tDCS plus 20 real mirror therapy sessions; first 10 sessions were combined	6.12 ± 1.88	3.27 ± 2.80	−2.85 ± 2.38	
3	Ferreira et al. (15)^a^	Neuropathic pain following traumatic brachial plexus injury	≥3 months	Sham tDCS and mirror therapy (8)	12	No available data	No available data	No available data	
				Real tDCS and mirror therapy (8)	12	No available data	No available data	No available data	
4	Soler et al. (17)	Neuropathic pain following spinal cord injury	≥6 months	Sham tDCS and control illusion (10)	10	7.1 ± 1.5	6.4 ± 1.9	−0.7 ± 1.71	
				Real tDCS and control illusion (10)	10	6.3 ± 2.0	6.1 ± 2.5	−0.2 ± 2.26	
				Sham tDCS and visual illusion (9)	10	7.2 ± 1.6	7.2 ± 1.5	0 ± 1.55	1.54
				Real tDCS and visual illusion (10)	10	7.5 ± 1.2	5.3 ± 1.4	−2.2 ± 1.30	
5	Soler et al. (16)^a^^,^^b^	Neuropathic pain following spinal cord injury	≥6 months	Control (no intervention) (65)	No treatment	31% ± 14	31% ± 14	0% ± 14	
				Real tDCS and visual illusion (65)	10	34% ± 16	25% ± 16	−9% ± 16	
6	Kumru et al. (18)^a^	Healthy subjects (14)		Real tDCS and visual illusion (14)	10	No available data	No available data	No available data	
		No neuropathic pain following spinal cord injury (20)		Real tDCS and visual illusion (20)	10	No available data	No available data	No available data	
		Neuropathic pain following spinal cord injury (18)	≥3 months	Real tDCS and visual illusion (20)	10	7.8 ± 0.9	4.9 ± 2.0	−2.9 ± 1.55	
7	López-Carballo et al. (22)^a^^,^^b^	Neuropathic pain following spinal cord injury (23)	≥3 months	Real tDCS and visual illusion with gestural control	10	14.4 ± 6.5	10.5 ± 7.3	−3.9 ± 6.9	
8	Lagueux et al. (24)	Complex regional pain syndrome	>3 months	Sham tDCS and graded motor imagery (11)	14 combined sessions: 10 sessions during first 2 weeks, then maintenance therapy for 4 more weeks	6.09 ± 1.51	4.91 ± 2.17	−1.18 ± 1.87	0.018
				Real tDCS and graded motor imagery (11)	14 combined sessions: 10 sessions during first 2 weeks, then maintenance therapy for 4 more weeks	5.95 ± 2.21	4.73 ± 2.69	−1.22 ± 2.46	

Only studies that performed ≥10 sessions were included in the table to allow a fair comparison.

All studies used the same tDCS montage, with the following considerations: in case of phantom pain, the anode was placed over the motor cortex contralateral to the amputated limb, and the cathode over the supraorbital area ipsilateral to the amputated limb. In neuropathic pain indications and complex regional pain syndrome, the anode was placed on the motor cortex contralateral to the painful side for patients with asymmetric pain and at the dominant hemisphere for patients with symmetric pain.

All the studies used a combination of tDCS and another non-pharmacological neuromodulatory approach. In all studies, the tDCS intensity was set to 2mA, except for Segal et al. (12), which used 1.5 mA. In all the studies, the tDCS duration was 20 min, except in Ferreira et al. (15), which used 30 min per session. The duration of the non-pharmacological neuromodulatory approaches ranged from 12 to 20 min, except for Ferreira et al. (15), which used 30 minutes per session. All studies conducted the combined therapy 5 times per week for 2 weeks, except Ferreira et al. (15), which conducted the therapy 3 times per week.

Change in pain was calculated as baseline pain minus pain 1 month after the end-of-treatment time-point, except in Gunduz et al. (13), Ferreira et al. (15), and Soler et al. (16), in which posttreatment pain intensity was measured at the end of treatment (and not 1 month later) because follow-up data at 1 month were unavailable. In these studies, effect size estimation is based on the pain intensity at the end of treatment. In López-Carballo et al. (22), change in pain was calculated with posttreatment data collected 15 days after end of treatment.

The effect size was calculated as the mean change in pain in real tDCS combined with a real neuromodulatory approach versus the mean change in the sham tDCS combined with a real neuromodulatory approach, divided by the pooled standard deviation, using the following formula $d=\frac{\left|M1-M2\right|}{\sqrt{\left(SD1^{2}+SD2^{2}\right)/2}}$. Hence, it provided an estimate to the effect of adding tDCS on top of the other neuromodulatory approach.

a Effect sizes were not calculated for the following reasons: In Soler et al. (16), Kumru et al. (18), and López-Carballo et al. (22), because one of two of study arms of interest was not included in the study design; in Ferreira et al. (15) the results were reported as medians of the McGill Pain Questionnaire, and because the means and standard deviations no were reported, the SES calculation was not possible.

b Soler et al. (16) and López-Carballo et al. (22) used the neuropathic pain symptoms inventory (NPSI). In Soler et al. (16), pain intensity was measured with NPSI as percentage of change.

In our study, at 1 month following the end of treatment, the analgesic effects were approximately twice as great as those found in the other studies. On the 0–10 scale, phantom pain intensity was reduced by an average of 6.2 points. Our study also showed much larger standardized effect size than did the other studies, except Soler et al. (17), which demonstrated similar effect size. Although Soler et al. (17) found modest average reductions in pain in the combined-treatment arm (−2.2 points on the 0–10 scale), they observed no change at all in the control arm. The lack of any pain reduction in the control produces a huge calculated effect size. In contrast, in our study, the reductions in pain in the 2 control arms were, as expected, in the magnitude of 2 and 3 points on the 0–10 scale in the mirror therapy alone and in the mirror therapy plus sham tDCS, respectively.

## 3. Discussion

To conclude, the data summarized in Table 2 support further investigation of our hypothesis. The analgesic effects of non-invasive brain stimulation combined with other neuromodulator treatments seem to be much stronger when the interventions are administrated at an early phase of the condition. Given that the comparison derived from Table 2 is descriptive rather than statistical, the results of this preliminary investigation should be regarded as a hypothesis generator. At the early onset of the painful condition—the acute stage—the abnormal neuroplasticity that is associated with the development of a chronic pain condition might not yet have been consolidated. By enrolling patients as early as possible after their pain develops, we might be at a favorable window of opportunity to counterbalance the abnormal neuroplasticity.

The rationale for our hypothesis assumes that after a longer period of pain, the abnormal neuroplasticity that is seen in various painful indications is already consolidated (71, 72) and might be resistant to changes. In contrast, at the acute phase, the central neuroplastic changes have not yet consolidated and are more easily reversed or even prevented. The importance of conducting neuroplasticity-related treatments soon after an injury is well-accepted in the rehabilitation arena, such as in treating post-stroke movement disorders (73). Interestingly, already 20 years ago, McCabe et al. (74) found that the analgesic effects of mirror therapy in complex regional pain syndrome are better when administrated at an early stage (< 8 weeks after onset of pain) than when administered later (1 year or more) (74).

Given the currently inadequate treatments for phantom limb pain and other chronic painful conditions, the healthcare field urgently needs therapeutic interventions to prevent chronicity. A clearer understanding of how maladaptive plasticity is related to the development of chronic pain and how neuromodulation interference at the acute stage can prevent it will pave the way toward a new era of pain treatment: clinical adoption of neuromodulation targeting dysfunctional networks. We encourage the relevant research community to test our hypothesis and to assess the benefits of combined neuromodulatory approaches at earlier time-points of symptoms duration, whenever possible, both in the field of pain and beyond.

## Data availability statement

The original contributions presented in the study are included in the article/supplementary material, further inquiries can be directed to the corresponding author.

## Author contributions

MA performed the literature search and contributed to writing and reviewing the manuscript. IW and RT conceptualization, writing, reviewing, and editing. All authors contributed to the article and approved the submitted version.
